# Effects of Motor Control-Based Interventions on Pain and Functional Outcomes in Bowed String Musicians: A Systematic Review

**DOI:** 10.3390/jcm15093326

**Published:** 2026-04-27

**Authors:** Aleksandra Adamik, Edyta Mikołajczyk, Jakub Szczechowicz

**Affiliations:** 1 Institute of Applied Sciences, Faculty of Motor Rehabilitation, University of Physical Culture, 31-571 Krakow, Poland; edyta.mikolajczyk@awf.krakow.pl (E.M.); jakub.szczechowicz@awf.krakow.pl (J.S.); 2 Specialized Hand Therapy Center, 31-871 Krakow, Poland

**Keywords:** motor control, musicians, musculoskeletal disorders

## Abstract

**Background/Objective:** Playing-related musculoskeletal disorders are highly prevalent among bowed string musicians and may impair performance and career longevity. This study aimed to evaluate the effects of motor control-based interventions on pain, functional outcomes, range of motion, and neuromuscular parameters in musicians playing bowed string instruments. **Methods:** A systematic review was conducted in accordance with PRISMA 2020 guidelines. PubMed, Scopus, Web of Science Core Collection, and Cochrane CENTRAL were searched from inception to October 2025, and the search was updated before resubmission to identify any newly published eligible studies. Eligibility screening, full-text assessment, data extraction, and risk-of-bias assessment were independently verified by a second reviewer. Risk of bias was assessed according to study design using RoB 2 for the randomized controlled trial and ROBINS-I for non-randomized interventional studies. **Results:** Four interventional studies met the inclusion criteria. Three studies reported improvements in pain-related outcomes or PRMD severity, and three reported improvements in functional outcomes. One study demonstrated improved cervical range of motion, whereas one study reported altered shoulder girdle muscle activation patterns with reduced playing comfort. Overall, the certainty of the available evidence was limited by small sample sizes, non-randomized designs, and risk of bias. **Conclusions:** Limited evidence suggests that motor control-based interventions may be associated with improvements in pain and playing-related function in bowed string musicians; however, the evidence base remains small and methodologically heterogeneous, and conclusions should be interpreted with caution.

## 1. Introduction

Playing bowed string instruments contributes to the development of specific musculoskeletal overload. Prolonged practice results in sustained asymmetric body posture and repeatedly performed movements of the upper limbs and neck. These occupational demands are reflected in the high prevalence of playing-related musculoskeletal disorders (PRMD). Symptoms most often involve the neck, shoulder, and upper extremity, frequently preventing participation in practice sessions or performances. In addition to pain, PRMD may reduce functional capacity, decrease playing comfort, and limit the ability to adopt an optimal playing posture [1].

Clinicians are rarely able to identify a single structural diagnosis in cases of PRMD. Symptoms are very often accompanied by biomechanically suboptimal movement strategies, resulting in inefficient neuromuscular coordination. Research on musicians who play bowed string instruments highlights a substantial gap in the literature regarding protective strategies and injury prevention in this population, which is exposed to markedly distinct biomechanical and neuromuscular demands [2]. Studies investigating motor patterns in these musicians have described increased coactivation and altered movement strategies during demanding performance or practice conditions. This suggests that impaired neuromuscular control may contribute to persistent symptoms and limitations in the ability to perform certain functional movements in specific subgroups of musicians [3].

Motor control-based interventions are widely used in the rehabilitation of musculoskeletal conditions, particularly in the management of movement-related disorders and neuromuscular dysfunction [4,5]. In the context of this review, motor control-based interventions are defined as approaches primarily targeting neuromuscular coordination, sensorimotor control, and task-specific movement efficiency, rather than isolated strength or conditioning programs. They aim to reduce the impact of modifiable factors such as impaired motor control, decreased neuromuscular coordination, or excessive antagonist activity. In bowed string musicians, this approach is typically implemented through cervical stabilization exercises, retraining of scapular movement, postural control training, and functional exercise. Emphasis is placed on improving endurance and neuromuscular coordination, with attention to education and the integration of movement patterns specific to the demands of the instrument [6,7,8]. Clinically, these strategies are attractive because they can be individualized to the musician’s instrument, technique, and performance demands, and may be integrated into practice routines without requiring prolonged playing cessation. However, despite their frequent clinical use, the available evidence remains limited and heterogeneous [9]. Studies vary in design, participant characteristics, and outcome measures, with inconsistent reporting across clinical (e.g., pain, PRMD severity) and mechanistic (e.g., biomechanical or neuromuscular) outcomes [10]. For example, a cervical stabilization program in university violinists was associated with improvements in pain, disability, and cervical impairments [7], while resistance training in professional string musicians was associated with improved playing-related outcomes and physical capacity [8]. Additionally, scapular taping during violin performance has been studied as a strategy to alter shoulder girdle muscle activation patterns without detriment to musical performance [6]. An individualized rehabilitation program with motor control elements has also shown improvements in PRMD-related outcomes in orchestral musicians, including a subgroup of string players [11]. Taken together, these findings suggest potential benefits but also highlight uncertainty regarding which components are most effective and which clinical and biomechanical outcomes are most responsive.

A focused synthesis is needed to help clinicians make better, evidence-informed decisions when rehabilitating bowed string musicians with PRMD. Specifically, it should clarify whether motor control-oriented interventions improve pain and playing-related function and, when available, whether they are accompanied by measurable biomechanical or neuromuscular changes. It should also highlight key gaps in the current literature to inform future trials and encourage more standardized, clinically relevant outcome reporting. The purpose of this systematic review was to evaluate the potential effects of motor control-based interventions on pain, functional outcomes, range of motion, and neuromuscular parameters in musicians playing bowed string instruments.

## 2. Materials and Methods

### 2.1. Protocol and Reporting Standard

This systematic review was conducted in accordance with the Preferred Reporting Items for Systematic Reviews and Meta-Analyses (PRISMA) 2020 guidelines. The review protocol was not prospectively registered in PROSPERO or another registry. However, the review question, eligibility criteria, outcomes of interest, and search approach were predefined before formal screening and data extraction. This lack of prospective registration is acknowledged as a methodological limitation. The absence of prospective registration may increase the risk of selective reporting, as methodological decisions could potentially be modified during the review process.

### 2.2. Eligibility Criteria- PICO Framework

Studies were eligible if they included musicians playing bowed string instruments (either at least 80% of the sample or a clearly reported subgroup analysis of string players), evaluated a motor control-oriented intervention, and reported at least one relevant outcome: pain, range of motion (ROM), electromyography (EMG), or playing-related function. Only interventional studies available as full-text articles in English were considered. Studies were excluded when data for bowed string musicians could not be separated from mixed instrument groups, when the intervention was not related to motor control, when the design was observational, review, or a case report, or when no clinical outcomes were reported (Table 1).

### 2.3. Search Strategy

The databases PubMed, Scopus, Web of Science Core Collection, and Cochrane CENTRAL were systematically searched from inception to October 2025. The search was updated prior to resubmission to ensure that no additional eligible studies published after this date were missed. Supplementary searching was performed using Google Scholar and by screening the reference lists of relevant articles. Google Scholar was searched as an additional source to identify potentially relevant studies not indexed in the primary databases. Due to its broad scope, only the first 200 results sorted by relevance were screened. No additional eligible records were identified through Google Scholar beyond those retrieved from the primary databases. The search strategy combined controlled vocabulary and free-text terms related to the population and intervention, including “violin”, “viola”, “cello”, “double bass”, “string musician”, “motor control”, “stabilization”, “exercise”, “postural training”, “electromyography”, “EMG”, “range of motion”, “ROM”, and “musculoskeletal pain”, using Boolean operators (AND/OR). No publication date restrictions were applied. Only articles published in English were considered. The complete search strategies for each database, including exact syntax, filters, and the date of the final search update, are provided in Supplementary Table S1.

### 2.4. Study Selection and Data Collection Process

**Figure 1:**
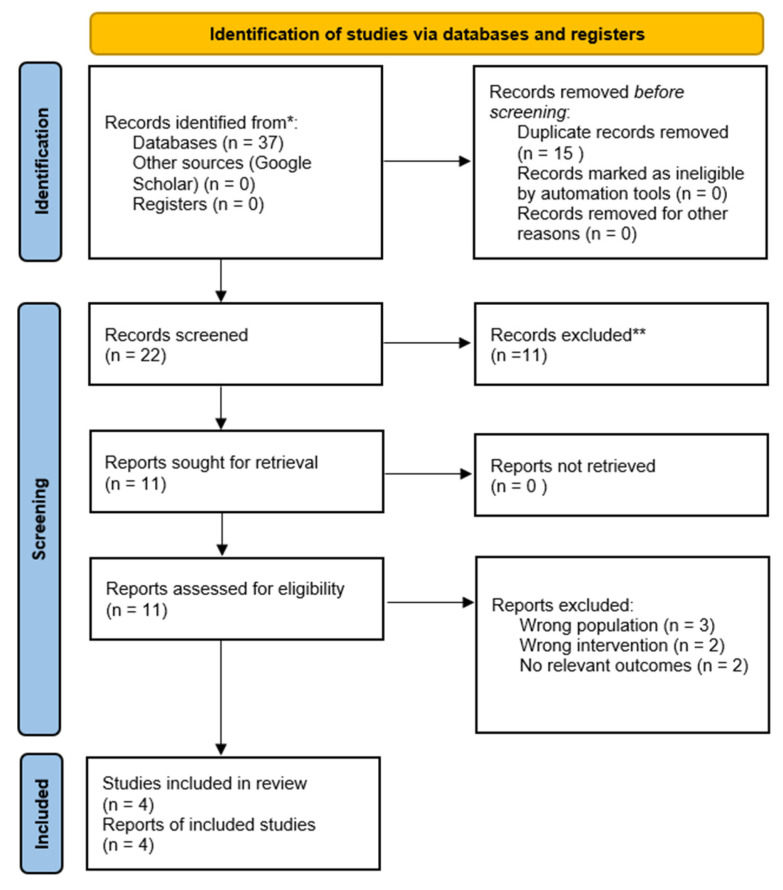
PRISMA 2020 flow diagram of the study selection process. * Records identified from databases represent the total number of articles retrieved from electronic database searches prior to deduplication. ** Records excluded after title and abstract screening based on predefined eligibility criteria (e.g., irrelevant topic, not meeting inclusion criteria, or duplicate records not previously removed).

Electronic searches were performed in PubMed, Scopus, Web of Science Core Collection, Cochrane CENTRAL, and Google Scholar. Records were imported and duplicates were removed before screening. Title and abstract screening was performed by one reviewer (A.A.) and independently verified by a second reviewer (E.M.). Full-text eligibility assessment was reviewed by both authors, and disagreements were resolved through discussion; when needed, consultation with the third author (J.S.) was used to reach consensus. A total of 37 records were identified from the primary databases (PubMed: 6; Cochrane CENTRAL: 6; Scopus: 16; Web of Science: 9); Google Scholar did not contribute any additional eligible studies beyond duplicate or ineligible records. After removal of duplicates (*n* = 15), 22 unique records remained. Following title and abstract screening, 11 records were excluded. Full-text articles were assessed for 11 publications, of which 7 were excluded due to wrong population (*n* = 3), wrong intervention (*n* = 2), or lack of relevant clinical outcomes (*n* = 2). Ultimately, 4 studies were included in the qualitative synthesis [6,7,8,11]. The study selection process is presented in Figure 1 (PRISMA 2020 flow diagram).

### 2.5. Data Extraction

Data extraction was performed using a standardized data extraction form. One reviewer (A.A.) extracted the data, and a second reviewer (E.M.) independently verified the extracted data, eligibility decisions, and risk-of-bias judgments against the full texts. The following information was extracted from each included study: authors and year of publication, country, study design, participant characteristics (sample size, age, sex, level of musical experience, and instrument), characteristics of the intervention (type of motor control or exercise-based intervention, duration, frequency, and use of biofeedback), comparator conditions, outcome measures, and main results. Biomechanical and neuromuscular outcomes, including motor control parameters and electromyographic or movement-based measures, were extracted when reported. Due to heterogeneity of study designs, interventions, and outcome measures, a meta-analysis was not considered appropriate and the findings were synthesized narratively.

### 2.6. Risk-of-Bias Assessment

Risk of bias was assessed according to study design. The randomized controlled trial was evaluated using the Cochrane Risk of Bias 2.0 (RoB 2) tool. The non-randomized interventional studies were assessed using the Risk Of Bias In Non-randomized Studies-of Interventions (ROBINS-I) tool. ROBINS-I judgments considered bias due to confounding, participant selection, classification of interventions, deviations from intended interventions, missing data, outcome measurement, and selection of the reported result. Risk-of-bias assessment was performed by one reviewer and independently verified by a second reviewer, with disagreements resolved by consensus.

## 3. Results

### 3.1. Study Selection

Of the 37 records identified from the primary databases, 22 publications remained after duplicate removal (*n* = 15). Google Scholar screening did not yield any additional eligible studies. Following title and abstract screening, 11 records were excluded. Full-text articles were retrieved and assessed for 11 studies, of which 7 were excluded for the reasons described above. Ultimately, 4 interventional studies involving musicians playing bowed string instruments were included in the qualitative synthesis [6,7,8,11].

### 3.2. Characteristics of Included Studies

The included studies involved sample sizes ranging from 8 to 35 participants. Two studies were conducted among violinists (students or professional musicians) [6,7], one study included professional bowed string musicians playing various instruments [8], and one study involved orchestral musicians with a subgroup analysis of bowed string players [11]. The interventions consisted of a cervical spine stabilization exercise program [7], scapular taping applied during violin performance [6], resistance training targeting postural endurance and capacity [8], and an individualized multimodal rehabilitation program incorporating motor control training and education [11]. Only one study was a randomized controlled trial; the remaining studies were non-randomized interventional studies. (Table 2).

### 3.3. Pain

Three studies [7,8,11] reported reductions in pain intensity or in the severity of playing-related musculoskeletal disorders (PRMD) following the intervention. These findings were observed in both violinists with neck pain and orchestral musicians with PRMD, although the magnitude and reporting of effects were heterogeneous and not always supported by complete effect estimates.

### 3.4. Range of Motion

One study [7] demonstrated improved cervical spine range of motion following a stabilization exercise program. The remaining studies did not report standardized range-of-motion outcomes.

### 3.5. EMG and Function

Ackermann et al. [6] observed altered shoulder girdle muscle activation patterns assessed by electromyography (EMG) following scapular taping during violin performance; however, these changes were accompanied by reduced playing comfort and no clear functional benefit. Three studies [7,8,11] reported improvements in functional outcomes, including disability, playing comfort, or participation-related measures.

### 3.6. Summary of Intervention Effects

Table 3 summarizes the clinical and biomechanical outcomes reported in the studies included in this systematic review. Pain and functional outcomes were most frequently reported across the included studies. The cervical stabilization program described by Kuo et al. [7] was associated with improvements in pain, disability, cervical range of motion, and selected cervical impairments. Lundborg and Grooten [8] reported improvements in strength, endurance, and some playing-related outcomes following resistance training, although reporting of effect estimates was incomplete. Roos and Roy [11] reported reductions in pain intensity and pain interference after a multimodal rehabilitation program in orchestral musicians.

Objective biomechanical and neuromuscular outcomes were reported less frequently. Only one study [6] assessed electromyographic outcomes and found altered shoulder girdle activation patterns during playing after scapular taping; however, these changes were associated with reduced playing comfort rather than improved performance. Similarly, only one study [7] reported standardized range-of-motion outcomes.

Taken together, the available studies suggest that motor control-oriented interventions may be associated with improvements in pain and playing-related function in this population. However, the evidence remains limited by small sample sizes, heterogeneity of interventions and outcomes, and substantial methodological limitations.

Clinical outcomes are presented separately from mechanistic outcomes to improve interpretability.

### 3.7. Risk of Bias Assessment of Included Studies

Risk of bias was assessed according to study design, using RoB 2 for the randomized controlled trial [12,13] and ROBINS-I for the non-randomized interventional studies. The single randomized controlled trial was judged as having some concerns of risk of bias, primarily related to deviations from intended interventions and limited blinding feasibility in an exercise-based context.

The three non-randomized studies were judged to be at serious risk of bias overall. The main concerns arose from potential confounding, non-random participant selection, absence of concealed allocation, lack of blinding, and incomplete or selective outcome reporting. These issues are inherent to small interventional studies without robust control conditions and limit confidence in the direction and magnitude of the observed effects.

Accordingly, the overall body of evidence should be interpreted cautiously. While the direction of findings was generally favorable for pain and functional outcomes, the methodological limitations of the included studies reduce certainty in these conclusions.

A summary of the RoB 2 and ROBINS-I assessments is presented in Table 4.

## 4. Discussion

The main finding of this systematic review is that motor control-oriented interventions may be associated with improvements in clinically relevant outcomes in bowed string musicians, particularly in terms of pain and playing-related function. Across the four included interventional studies, improvements in clinical outcomes were reported more consistently than changes in mechanistic outcomes, which were assessed less frequently [5,7,8,11]. Importantly, these findings should be interpreted with caution due to the limited number of included studies, their small sample sizes, and the predominance of non-randomized designs. Although the direction of effects was generally consistent for pain and functional outcomes, the methodological limitations of the included studies reduce confidence in the magnitude and reproducibility of these effects. Therefore, the current evidence should be considered preliminary rather than definitive. From a clinical perspective, these findings align with current rehabilitation trends that emphasize modifiable contributors to symptoms (e.g., load tolerance, neuromuscular coordination, and task-specific movement efficiency) rather than isolated structural explanations [14]. Performing arts medicine increasingly advocates approaches integrating education, graded exposure to performance demands, and restoration of efficient postural and scapulothoracic control, particularly for sustained asymmetric tasks typical of bowed string performance [15,16]. The cervical stabilization program with pressure biofeedback in university violinists was associated with improvements in pain, disability, cervical ROM, and craniovertebral angle, supporting the clinical rationale for targeting cervical sensorimotor control in musicians with neck pain [7]. This is consistent with broader neck pain rehabilitation frameworks emphasizing deep cervical muscle function and sensorimotor retraining to improve symptoms and disability, although direct extrapolation should be cautious given the musician-specific task demands [17,18]. Resistance training in professional string musicians with PRMD was associated with reductions in pain and improvements in upper-limb function (DASH), suggesting that increasing physical capacity and postural endurance may improve tolerance to prolonged playing loads [8]. This mirrors the growing focus in musculoskeletal practice on graded strengthening to improve tissue and task tolerance, thereby reducing symptom provocation during repetitive or sustained activities [19,20]. Importantly, motor control-oriented rehabilitation in musicians may be delivered not only through low-load stabilization, but also through functional strengthening that improves coordination and endurance under performance-relevant loads. Across the included studies, most reported improvements in pain-related and functional outcomes, although the magnitude and consistency of these effects varied. In particular, the cervical stabilization program with biofeedback reported improvements in pain, disability, and cervical impairments [7], while resistance training in professional string musicians was associated with reductions in PRMD severity and improved functional status [8]. However, resistance training should not be considered a purely motor control-based intervention, and its effects may be mediated by improvements in physical capacity rather than changes in motor control per se. These findings may be clinically relevant because they align with contemporary rehabilitation paradigms emphasizing not only symptom reduction but also improved load tolerance and task capacity during sustained and repetitive occupational activities. In musicians, this translates to improved tolerance for practice duration, reduced symptom provocation during rehearsals, and improved participation in performance-related activities.

The mechanisms underlying the observed improvements remain uncertain. Changes in pain and function may reflect multiple interacting factors, including improved load tolerance, increased physical capacity, altered movement strategies, or non-specific effects of structured intervention [21]. Importantly, in bowed string musicians, functional strengthening can be conceptualized as motor control training when it targets coordinated postural endurance and task-specific movement efficiency rather than isolated strength gains. However, it is important to note that causal interpretation remains limited. In particular, the multimodal nature of some interventions makes it difficult to isolate the specific contribution of motor control components from other elements such as education, load management, or general exercise. This reflects a broader challenge in rehabilitation research, where complex interventions are frequently applied, but their individual components are not easily separable.

The scapular taping study suggests that altering proprioceptive input may influence muscle activation patterns; however, it was associated with reduced playing comfort and no clear functional benefit, limiting its clinical applicability as a standalone intervention [6]. Clinically, this supports the use of taping primarily as a short-term cueing strategy, potentially facilitating scapular control or reducing excessive upper trapezius dominance during demanding playing tasks rather than as a capacity-building intervention. This distinction is relevant because current clinical guidance increasingly emphasizes active approaches and graded exposure over passive modalities for sustained functional change [22]. Therefore, when taping is used, it may be best integrated with active scapular retraining and progressive endurance work rather than applied in isolation.

Multimodal interventions reflect real-world clinical practice but limit causal interpretation, as the specific contribution of motor control components cannot be clearly isolated. Specifically, it remains unclear whether observed improvements are driven by motor control elements, education and behavior change, or non-specific effects of structured care [9]. This issue reflects a broader methodological challenge in rehabilitation research, and current reporting frameworks emphasize the need for detailed intervention descriptions (e.g., progression criteria, adherence, co-interventions) to support replication and clinical translation [23]. Future studies in musician rehabilitation should therefore provide transparent reporting of intervention content and dose, and where feasible, consider study designs that better isolate component effects.

A notable gap identified in this review is the limited use of objective biomechanical and neuromuscular outcomes. Only one study reported standardized cervical ROM changes [7], and only one assessed EMG outcomes [6]. This limits mechanistic conclusions, as improvements in pain and function could reflect multiple pathways, including improved load management, increased physical capacity, altered movement strategies, reduced co-contraction, or contextual (non-specific) effects [12,13,24]. Given current trends toward mechanism-informed rehabilitation, future trials should integrate validated clinical outcomes (pain and disability) with objective measures that reflect playing-specific demands, such as task-based EMG, movement analysis during standardized playing tasks, and endurance-related metrics that capture tolerance to sustained postures [25,26]. Such measures would strengthen causal inference and help determine whether improvements in symptoms are accompanied by changes in neuromuscular coordination during performance.

Importantly, clinical outcomes (pain and function) were reported more consistently than mechanistic outcomes (e.g., ROM, EMG), which limits conclusions regarding underlying physiological mechanisms.

Overall, the available evidence suggests potential benefits of motor control-oriented interventions in bowed string musicians; however, the small number of studies, methodological heterogeneity, and risk of bias substantially limit confidence in these findings. Therefore, conclusions should remain cautious, and clinical application should be individualized rather than protocol-driven [7,8,11].

The limited number of included studies reflects the scarcity of interventional research specifically investigating motor control-based rehabilitation in bowed string musicians rather than limitations of the search strategy. Despite conducting a comprehensive and updated search, no additional eligible studies were identified, confirming the limited availability of research in this specific area. This highlights a substantial gap in the literature and emphasizes the need for further high-quality studies.

This population represents a highly specialized group with distinct neuromuscular and biomechanical demands, and interventional trials remain challenging due to small sample sizes and heterogeneity in instruments, playing techniques, and professional status. As a result, the available evidence base remains limited and methodologically diverse.

Several methodological limitations of this review should also be acknowledged. First, the review was not prospectively registered, which may increase the risk of selective reporting. Second, although independent verification by a second reviewer was introduced during the revision process, the review was not originally designed with duplicate independent screening and data extraction. Third, the evidence base consisted of only four studies with heterogeneous interventions and outcomes. Finally, three of the four included studies were non-randomized and judged to be at serious risk of bias using the ROBINS-I tool. These factors substantially limit the certainty of the conclusions.

Given these limitations, the findings of this review should be interpreted cautiously. The available evidence suggests that motor control-based interventions may improve clinically relevant outcomes, particularly pain severity and playing-related function; however, the strength of these conclusions is limited by the small number of studies, their methodological heterogeneity, and the risk of bias. Future research should prioritize well-designed randomized controlled trials with larger samples, standardized intervention protocols, and combined clinical and objective neuromuscular outcome measures to improve the evidence base and guide clinical decision-making.

Therefore, these findings should not yet be interpreted as a basis for standardized clinical protocols, and clinical application should remain individualized and guided by patient-specific factors.

## 5. Conclusions

Motor control-oriented interventions may be associated with improvements in pain and playing-related function in musicians who play bowed string instruments. The most consistent findings relate to reductions in pain and improvements in functional outcomes, whereas improvements in cervical range of motion and changes in muscle activation patterns have been reported only in single studies. However, the available evidence is limited by the small number of studies, their methodological heterogeneity, and the predominance of non-randomized designs with associated risk of bias. Therefore, these findings should be interpreted with caution. Further high-quality research with larger samples and more standardized intervention protocols is needed.

## Figures and Tables

**Table 1:** PICO framework.

Population (P): Musicians playing bowed string instruments (violin, viola, cello, double bass), including mixed samples if data for string players were reported separately.
Intervention (I): Motor control-based interventions targeting neuromuscular coordination, sensorimotor control, postural control, or task-specific movement efficiency (e.g., cervical stabilization, postural training, neuromuscular retraining), with or without biofeedback.
Comparison (C): No intervention, usual practice, or alternative exercise or rehabilitation interventions.
Outcomes (O): Clinical outcomes: pain intensity, PRMD severity, functional performance, playing-related disability or comfort. Mechanistic outcomes: range of motion (ROM), electromyography (EMG), neuromuscular or biomechanical parameters

EMG—electromyography.

**Table 2:** Characteristics of included studies.

Study	Design	Participants	Intervention	Comparator	Outcomes	Main Results	Conclusions
Kuo et al., 2020 [7]	Quasi-experimental (single-group pre–post study)	*n* = 20 university violinists (10 F/10 M), mean age 21.2 ± 3.2 yrs, chronic nonspecific neck pain	Cervical stabilization exercise program with pressure biofeedback; 6 weeks, 5×/week, 20 min/session	Baseline comparison	Pain (NRS); disability (NDI); cervical ROM; muscle endurance; proprioception; craniovertebral angle	↓ Pain (NRS); ↓ disability (NDI); ↑ cervical ROM (except flexion); ↑ muscle endurance; ↑ proprioception	Cervical stabilization with biofeedback may improve pain, disability, and neuromuscular control
Ackermann et al., 2002 [6]	Experimental	Professional violinists; *n* = 8	Scapular taping during performance tasks	No taping	Upper trapezius EMG; performance parameters; playing comfort	↑ Upper trapezius EMG (+49–60%); ↓ playing comfort; no improvement in performance	Scapular taping altered muscle activation but reduced comfort and did not improve performance
Lundborg & Grooten, 2018 [8]	Prospective intervention study	Professional string musicians with PRMDs	Progressive resistance training; 12 weeks, 2×/week	Baseline comparison	Pain (NRS); function (DASH); strength; endurance; PRMD severity	↑ Strength (11–19%); ↑ endurance (25%); ↓ PRMD severity (29–59%); inconsistent effects on pain and function	Resistance training may improve physical capacity and selected PRMD-related outcomes
Roos & Roy, 2018 [11]	RCT	Student and professional orchestral musicians with PRMDs	Multimodal rehabilitation program; 6 weeks, 3×/week	Usual care	Pain intensity; pain interference; PRMD prevalence/frequency	↓ Pain intensity; ↓ pain interference; no effect on PRMD prevalence/frequency	Multimodal rehabilitation reduced pain intensity and pain interference

*PRMD—playing-related musculoskeletal disorders; ROM—range of motion; EMG—electromyography; NDI—Neck Disability Index; NRS—Numeric Rating Scale; DASH—Disabilities of the Arm, Shoulder and Hand; RCT—randomized controlled trial.*

**Table 3:** Summary of clinical and biomechanical outcomes of included studies.

Study	Pain/PRMD	Range of Motion (ROM)	EMG Outcomes	Functional Outcomes
Kuo et al., 2020 [7]	↓ Neck pain (clinically significant)	↑ Cervical ROM	Not assessed	↑ NDI; improved functional status
Ackermann et al., 2002 [6]	Not assessed	Not assessed	Altered shoulder girdle activation	↓ Playing comfort; no performance benefit
Lundborg & Grooten, 2018 [8]	↓ PRMD severity	Not assessed	Not assessed	↑ Playing comfort; ↑ strength
Roos & Roy, 2018 [11]	↓ PRMD severity	Not assessed	Not assessed	↑ Functional performance; ↑ participation

*PRMD—playing-related musculoskeletal disorders; ROM—range of motion; EMG—electromyography; NDI—Neck Disability Index.*

**Table 4:** Risk-of-bias assessment of included studies using RoB 2 and ROBINS-I.

Study	Design	Tool	Overall Risk of Bias	Main Concerns
Kuo et al., 2020 [7]	Quasi-experimental	ROBINS-I	Serious	Confounding, lack of control group, selection bias, outcome measurement
Ackermann et al., 2002 [6]	Experimental	ROBINS-I	Serious	Small sample size, selection bias, lack of blinding, incomplete outcome reporting
Lundborg & Grooten, 2018 [8]	Prospective intervention	ROBINS-I	Serious	Confounding, no randomization, baseline comparison only, reporting limitations
Roos & Roy, 2018 [11]	Randomized controlled trial	RoB 2	Some concerns	Deviations from intended interventions, limited blinding

## Data Availability

No new data were created or analyzed in this study. Data sharing is not applicable.

## Supplementary Materials

The following supporting information can be downloaded at: https://www.mdpi.com/article/10.3390/jcm15093326/s1, Table S1: Full search strategies for each database; PRISMA 2020 Checklist [24].
