# Supplementary material for: Effects of Motor Control-Based Interventions on Pain and Functional Outcomes in Bowed String Musicians: A Systematic Review
Source: J Clin Med. 2026 Apr 27;15(9):3326. doi: 10.3390/jcm15093326 (PMC13164197; doi:10.3390/jcm15093326)
Supplement: Supplementary file 1 [file jcm-15-03326-s001.zip › jcm-4276847-supplementary.pdf]

Supplementary Table S1. Full search strategies for each database.

Database	Search strategy	Filters	Date of last search
**PubMed**	("violin"[Title/Abstract] OR "viola"[Title/Abstract] OR "cello"[Title/Abstract] OR "double bass"[Title/Abstract] OR "string musician"[Title/Abstract]) AND ("motor control"[Title/Abstract] OR "stabilization"[Title/Abstract] OR "exercise"[Title/Abstract] OR "postural training"[Title/Abstract]) AND ("pain"[Title/Abstract] OR "musculoskeletal"[Title/Abstract] OR "EMG"[Title/Abstract] OR "electromyography"[Title/Abstract] OR "range of motion"[Title/Abstract] OR "ROM"[Title/Abstract])	English	October 2025 (updated April 2026)
**Scopus**	TITLE-ABS-KEY (violin OR viola OR cello OR "double bass" OR "string musician") AND TITLE-ABS-KEY ("motor control" OR stabilization OR exercise OR "postural training") AND TITLE-ABS-KEY (pain OR musculoskeletal OR EMG OR electromyography OR "range of motion" OR ROM)	English	October 2025 (updated April 2026)
**Web of Science**	TS=(violin OR viola OR cello OR "double bass" OR "string musician") AND TS=("motor control" OR stabilization OR exercise OR "postural training") AND TS=(pain OR musculoskeletal OR EMG OR electromyography OR "range of motion" OR ROM)	English	October 2025 (updated April 2026)
**Cochrane CENTRAL**	(violin OR viola OR cello OR "double bass" OR "string musician") AND ("motor control" OR stabilization OR exercise OR "postural training") AND (pain OR musculoskeletal OR EMG OR electromyography OR "range of motion" OR ROM)	None applied	October 2025 (updated April 2026)
**Google Scholar**	"violin" AND "motor control" AND pain	First 200 results sorted by relevance	October 2025 (no additional eligible studies identified)
